# Diagnostic Performance of PET/CT Using 18F-FACBC in Prostate Cancer: A Meta-Analysis

**DOI:** 10.3389/fonc.2019.01438

**Published:** 2020-01-10

**Authors:** Xu Bin, Shan Yong, Qing-Fang Kong, Sun Zhao, Guang-Yuan Zhang, Jian-Ping Wu, Shu-Qiu Chen, Wei-Dong Zhu, Ke-Hao Pan, Mu-Long Du, Ming Chen

**Affiliations:** ^1^Department of Urology, Affiliated Zhongda Hospital of Southeast University, Nanjing, China; ^2^The Second People's Hospital of Taizhou, Taizhou, China; ^3^Department of Nosocomial Infection, Affiliated Zhongda Hospital of Southeast University, Nanjing, China; ^4^Jiangsu Key Laboratory of Cancer Biomarkers, Prevention and Treatment, Department of Environmental Genomics, Collaborative Innovation Center for Cancer Personalized Medicine, Nanjing Medical University, Nanjing, China; ^5^Department of Biostatistics Center for Global Health, School of Public Health, Nanjing Medical University, Nanjing, China

**Keywords:** prostate cancer, PET, CT, 18F-fluciclovine(FACBC), meta-analysis

## Abstract

**Background:** Diagnostic performance of PET/CT using 18F-fluciclovine (18F-FACBC) in patients with prostate cancer (PCa) has been evaluated in only a few studies. There is no consensus on the diagnostic value of 18F-FACBC PET/CT in PCa recurrence or metastasis (except for bone metastasis), the primary diagnosis of the lesion. Hence, a meta-analysis was conducted to evaluate the performance of 18F-FACBC PET/CT.

**Methods:** The literature published from June 2015 to June 2019 on using 18F-FACBC PET/CT for the diagnosis of PCa was retrieved from PubMed and EMBASE. Pooled sensitivity (Sen), specificity (Spe), positive and negative likelihood ratios (LR+ and LR–), area under the curve (AUC), and diagnostic odds ratio (DOR) of 18F-FACBC PET/CT in patients with PCa were calculated. An SROC map was made, and a meta-regression analysis was carried out. A Fagan plot and likelihood ratio dot plot were drawn. Sensitivity and funnel plot analysis were made. Meta-disc, Review Manager 5.3, and STATA 13 were used for the meta-analysis.

**Results:** A total of nine articles met the strict criteria for diagnostic meta-analysis, which included 363 patients and 345 lesions. Pooled Sen, Spe, LR+, LR–, DOR were 0.88, 0.73, 3.3, 0.17, and 20, respectively. Lesions detected on the PET/CT image included primary lesions and metastases. For the lesion, the doctors considered the abnormal part as a lesion on the PET/CT image by their own experience and expertise, including primary lesions and metastases. For the patient, patients who participated in the trial can be diagnosed as PCa through 18F-FACBC.

**Conclusion:** This study comprehensively evaluated the diagnostic value of 18F-FACBC PET/CT on PCa. Our analysis suggests that 18F-FACBC PET/CT is a valuable agent in diagnosing PCa. More studies are needed for further validation.

## Introduction

At present, the incidence of prostate cancer (PCa) has increased significantly and, in the United States, has surpassed that of lung cancer, becoming the primary cancer to influence men's health (1). According to the American Cancer Society, there were ~174,650 new PCas in the United States in 2019 and 31,620 deaths from this disease (1). By 2000, the incidence of PCa was 4.55 per 100,000 males, and the case fatality rate was 5.59 per 100,000 people (2). In recent years, with the development of medical technology, its detection rate has increased year by year (3). Therefore, finding the right diagnosis followed by appropriate treatment is especially important.

The diagnosis of the PCa mainly depends on prostate-specific antigen (PSA) levels, needle biopsy, and imaging examination. In the past 20 years, with the promotion of PSA measurement, the discovery of people who have early PCa has increased significantly (4). However, PSA is not a specific marker for PCa, and it can be expressed not only by PCa cells but also by normal prostate cells and prostatic hyperplasia cells (5). A variety of factors and diseases can affect the level of PSA that leads to the release of excessive PSA into the blood and its fluctuations, such as benign prostatic hyperplasia, prostatitis, and activities (6). Computed tomography (CT) mainly observes the shape and density of the prostate. However, early PCa is limited to the capsule, and some tumors are of equal density with and are indistinguishable from normal glandular tissues. CT diagnosis is therefore difficult. PET can generate a fused function and morphological image that can improve the diagnostic accuracy (7). However, the current imaging agents, such as 18F-FDG and 67Ga-citrate, are non-bone-specific imaging agents. It is difficult to accurately locate the lesion. In some cases, previous bone lesions detected by 18F-FDG were mistaken for soft tissue (8). These techniques have low sensitivity for imaging bone metastases.

Various new technologies have been applied clinically, which has broadened the horizon of diagnosis and treatment of PCa. At the same time, many new problems have emerged because of the characteristics of PCa. At present, many studies on the diagnosis of PCa are still in the exploratory stage, and no ideal imaging agent has been found so far. This is an urgent problem to be solved. Fluorine 18 fluciclovine (18F-FACBC) is a radiolabeled amino acid analog that takes advantage of the amino acid transport upregulation in several types of cancer cells. FACBC is the only one that can move in and out of the PCa cell compared with 18F-FDG, gadolinium 68 (68 Ga), and carbon 11 choline (C-11) (9). FACBC is taken up to a greater extent in PCa cells than in surrounding normal tissue, providing an opportunity for its use in diagnosing this common cancer. There are few studies investigating 18F-FACBC with PET/CT. We therefore conducted a meta-analysis to evaluate the diagnostic value of 18-FACBC PET/CT in PCa.

## Methods

This meta-analysis was carried out according to the QUADAS-2 (Quality Assessment of Diagnostic Accuracy Studies-2) standard that describes the items required for meta-analyses of diagnostic accuracy studies. The QUADAS-2 tool is a diagnostic accuracy test, consisting of four parts: the choice of case, the test to be evaluated, the gold standard, and the case process (10). All components were assessed for risk of bias.

### Search Strategy

The authors performed a comprehensive computer literature search of PubMed and the EMBASE database to find studies on the diagnostic accuracy of 18F-FACBC PET/CT in patients with PCa, with a search time ending in June 2019. This search algorithm based on a combination of terms was created and used: (A) “18F-FLUCICLOVINE” AND (B) “PET/CT” AND (C) “PROSTATE CANCER.” No beginning date limit or language restrictions were used. References of the retrieved articles were also screened to search for possible additional articles.

### Study Selection

The exclusion criteria for the systematic review were: (a) articles not within the field of interest; (b) editorials or letters, review articles, comments, conference proceedings; and (c) case reports. All of the coauthors independently screened the abstracts of the retrieved articles. Subsequently, the researchers independently reviewed the full text of the selected articles to assess their eligibility for inclusion. Disagreements were solved through a consensus meeting among all coauthors.

### Data Extraction

For each selected study, information was collected on basic study characteristics (authors, year of publication, study design), patient characteristics (age, PSA), and data on diagnostic accuracy on a per patient-based analysis (including true-positive and true-negative findings, false-positive and false-negative findings, sensitivity, specificity, positive and negative predictive values, and accuracy).

### Statistical Analysis

Sensitivity and specificity, positive and negative likelihood ratios (LR+ and LR–), area under the curve (AUC), and diagnostic odds ratio (DOR) of visual analysis of 18F-FACBC PET/CT in patients with PCa were obtained from studies. Between-study statistical heterogeneity was assessed using *I*^2^ and the Cochrane Q test on the basis of the random-effects analysis. The diagnostic odds ratio was made as the effect amount.

The meta-regression and subgroup analysis of 18F-FACBC are shown in **Figure 5**, divided into four subgroups according to whether the articles analyzed patients or lesions, whether the group size was >50, whether PSA was >5 ng/ml, and whether the study was retrospective or prospective.

## Results

### Literature Search

A total of 43 records were found in the comprehensive computer literature search of the databases, and after the abstracts were screened, 35 records were excluded. There were 15 articles not in the field of interest, six were reviews, and seven were reports. The date of seven articles was unavailable. After reading the title, abstract, and full text, a total of nine articles were included. There were 363 PCa patients and 345 lesions included in the quantitative meta-analysis. The characteristics of the studies and diagnostic accuracy data from these articles are shown in Table 1.

**Table 1:** Study and patient characteristics.

**References**	**Year**	**Size**	**Age**	**PSA**	**Study type**	**Date type**	**Dose**	**TP (%)**	**FP (%)**	**FN (%)**	**TN (%)**
Nanni et al. (11)	2015	50	67	3.2	Retrospective	Lesion-based	374 ± 19 MBq	89	31	11	69
Miller et al. (12)	2017	110	67.4	5.87 ng/ml	Retrospective	Patient-based	370 MBq	91	9	51	49
Calais et al. (13)	2018	10	70.8	1.0/1.1 ng/ml	Retrospective	Patient-based	371 MBq	20	63	80	37
Selnæs et al. (14)	2018	28	66.2	14.6 ng/ml	Retrospective	Lesion-based	370 MBq	40	60	0	100
						Patient-based		30	70	0	100
Suzuki et al. (15)	2019	28	67.3	88.6 ng/ml	Retrospective	Patient-based	185 MBq	57	43	15	85
Akin-Akintayo et al. (16)	2018	24	70.8	8.5 ng/ml	Prospective	Lesion-based	370 MBq	95	5	47	53
Elschot et al. (17)	2018	28	66	5.2 ng/ml	Prospective	Lesion-based	/	95	5	11	89
Jambor et al. (18)	2018	32	65	12.0 ng/ml	Retrospective	Patient-based	370 MBq	84	16	4	96
Odewole et al. (19)	2016	53	67.5	7.2 ng/ml	Retrospective	Lesion-based	358 MBq	70	30	23	77

### Qualitative Analysis

**Figure 1:**
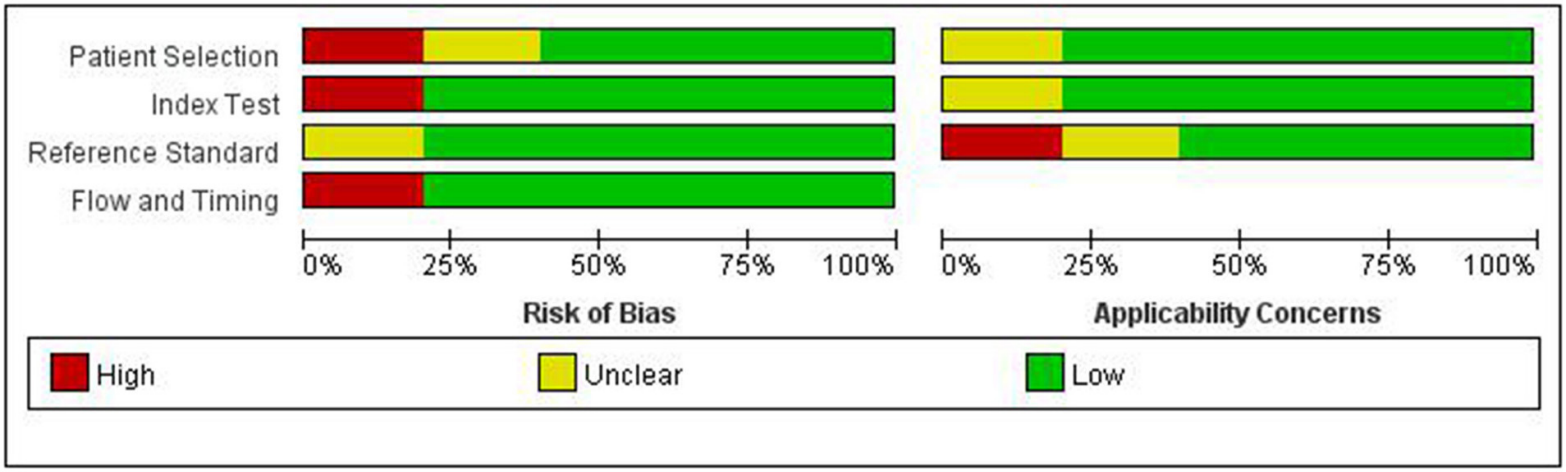
Summary of risk of bias and applicability concerns.

The quality of the articles included was not completely satisfactory. Some articles did not detail gold standards and patient selection. The research quality evaluation is shown in Figure 1.

#### Basic Study and Patient Characteristics

Screening the PubMed and EMBASE, nine articles evaluating the diagnostic performance of 18F-FACBC PET/CT in patients with PCa were selected. Seven of the selected articles were retrospective studies published from 2015 to 2019; the other two articles were prospective studies. The mean age of the patients included in these studies ranged from 66.2 to 70.8 years. The PSA of the patients included in the studies ranged from 1.0 to 14.6 ng/ml.

#### Meta-Analysis

**Figure 2:**
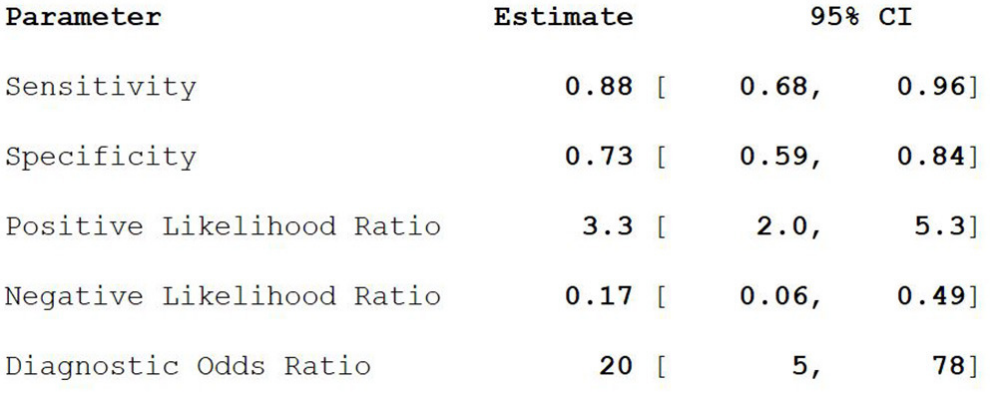
The combined statistics CI, confidence interval.

Nine studies including 363 PCa patients and 345 lesions were selected for the meta-analysis. Results of the meta-analysis are presented in Figure 2.

**Figure 3:**
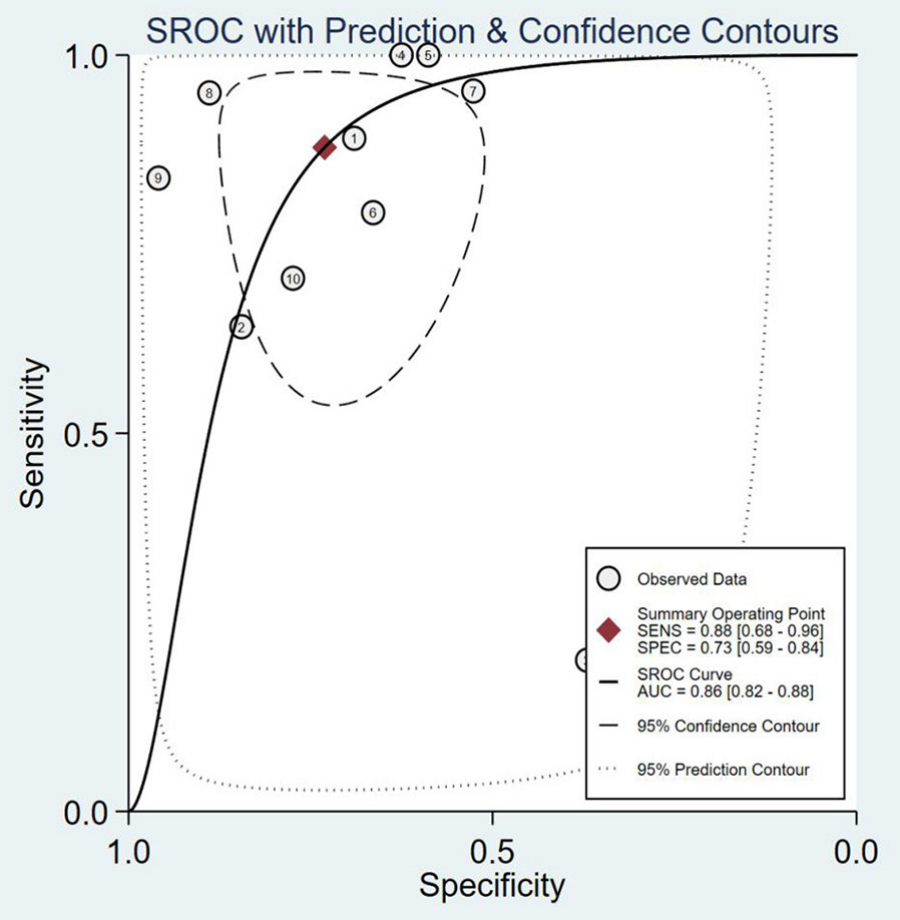
SROC curves of 18F-fluciclovine in PET/CT for diagnosis of prostate cancer. SENS, sensitivity; SPEC, specificity; AUC, area under the curve.

**Figure 4:**
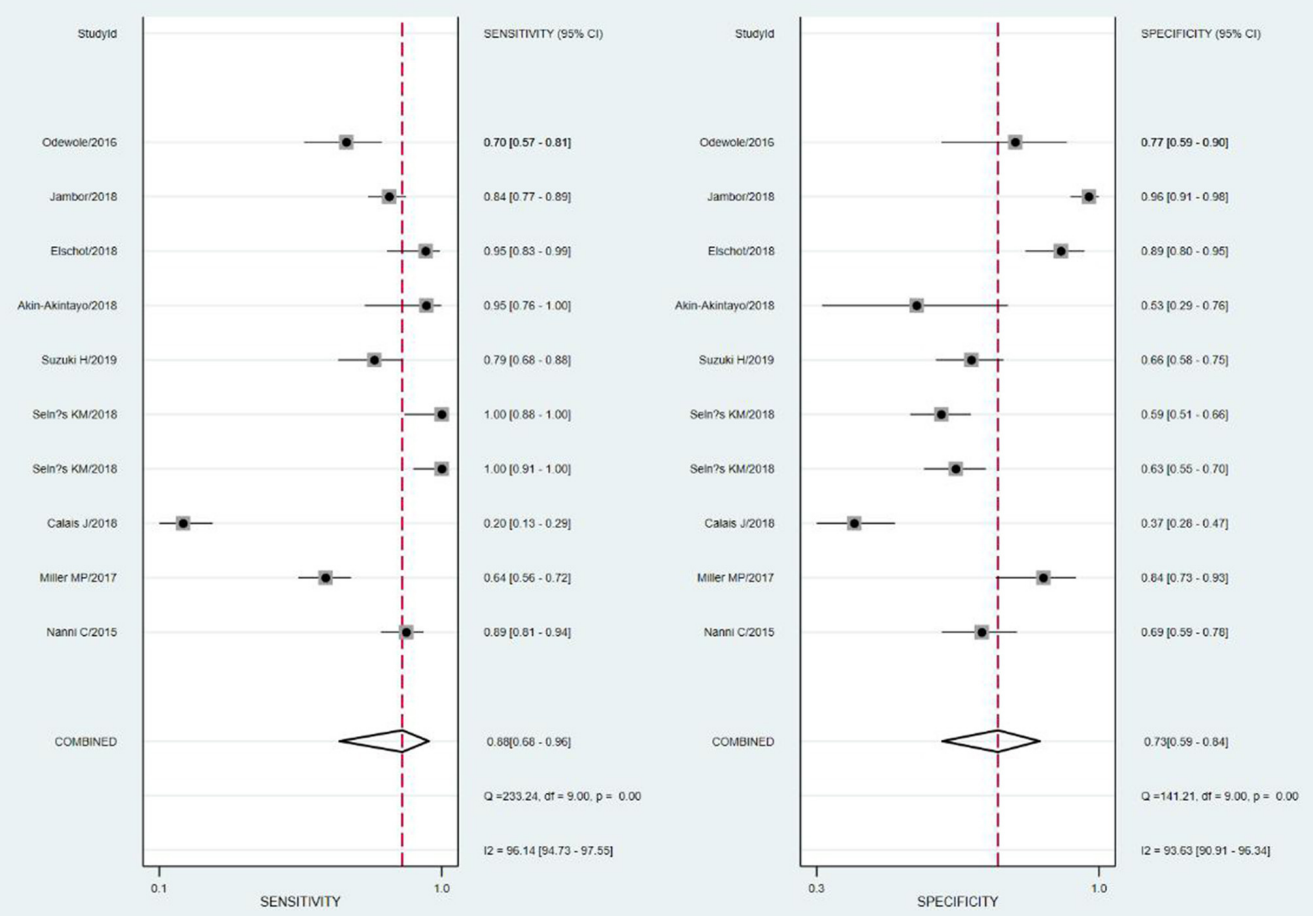
Forest map of 18F-fluciclovine PET/CT for diagnosis of prostate cancer.

The SROC curve and the forest map of 18F-FACBC PET/CT are shown in Figures 3, 4, respectively.

#### Heterogeneity Analysis

The results show that the Spearman coefficient is 0.957 (*P* < 0.001), indicating that there is no obvious heterogeneity. A fixed-effects model was used for statistical pooling of data on sensitivity and specificity. The observed SROC curve does not appear to be “shoulder arm shape,” suggesting that there is no threshold effect.

**Figure 5:**
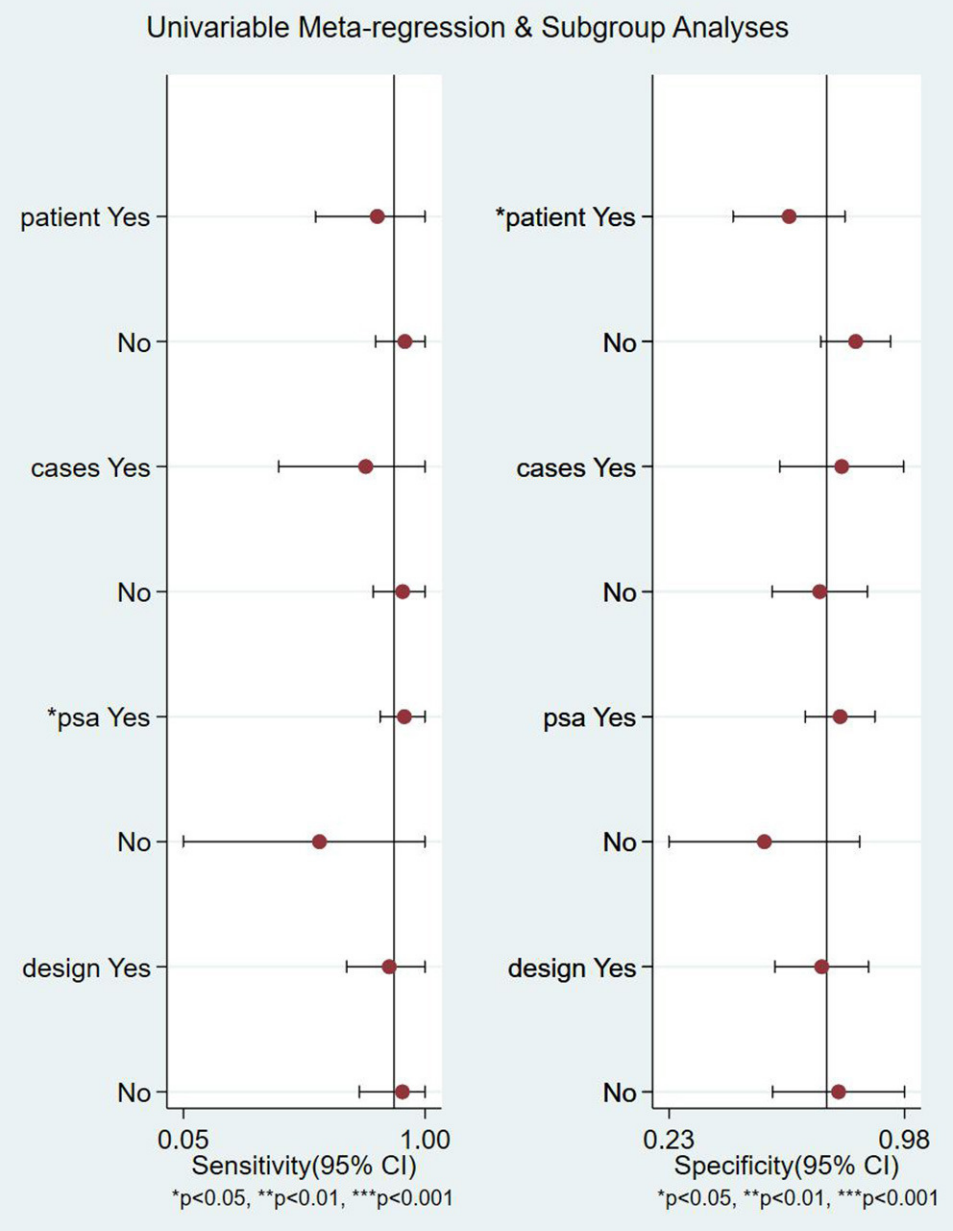
Meta-regression and subgroup analyses of studies. CI, confidence interval; PSA, prostate-specific antigen.

There is no significant heterogeneity in the sensitivity of the included studies. A meta-regression and subgroup analysis were performed to explore sources of heterogeneity in the included studies. It showed that no definite variable was the source of heterogeneity in the current meta-analysis (Figure 5).

#### Sensitivity Analysis

**Figure 6:**
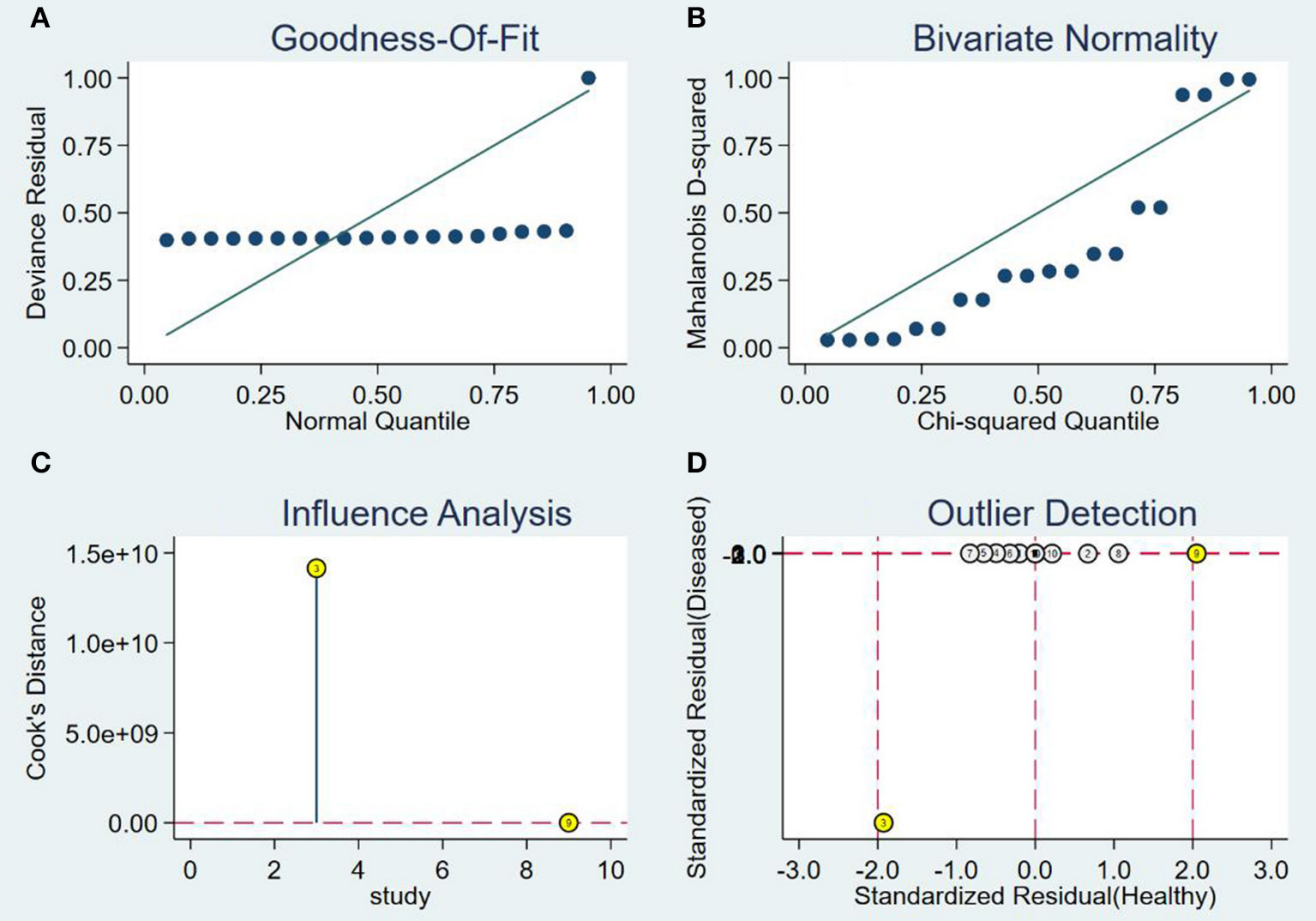
Sensitivity analysis of studies. **(A)** Goodness-of-fit, **(B)** bivariate normality, **(C)** influence analysis, and **(D)** outlier detection.

The SROC curve is analyzed by removing the included studies one by one. It is shown in Figure 6. The results showed that there were no significant changes, suggesting that this meta-analysis was integrated.

#### Clinical Application Analysis

**Figure 7:**
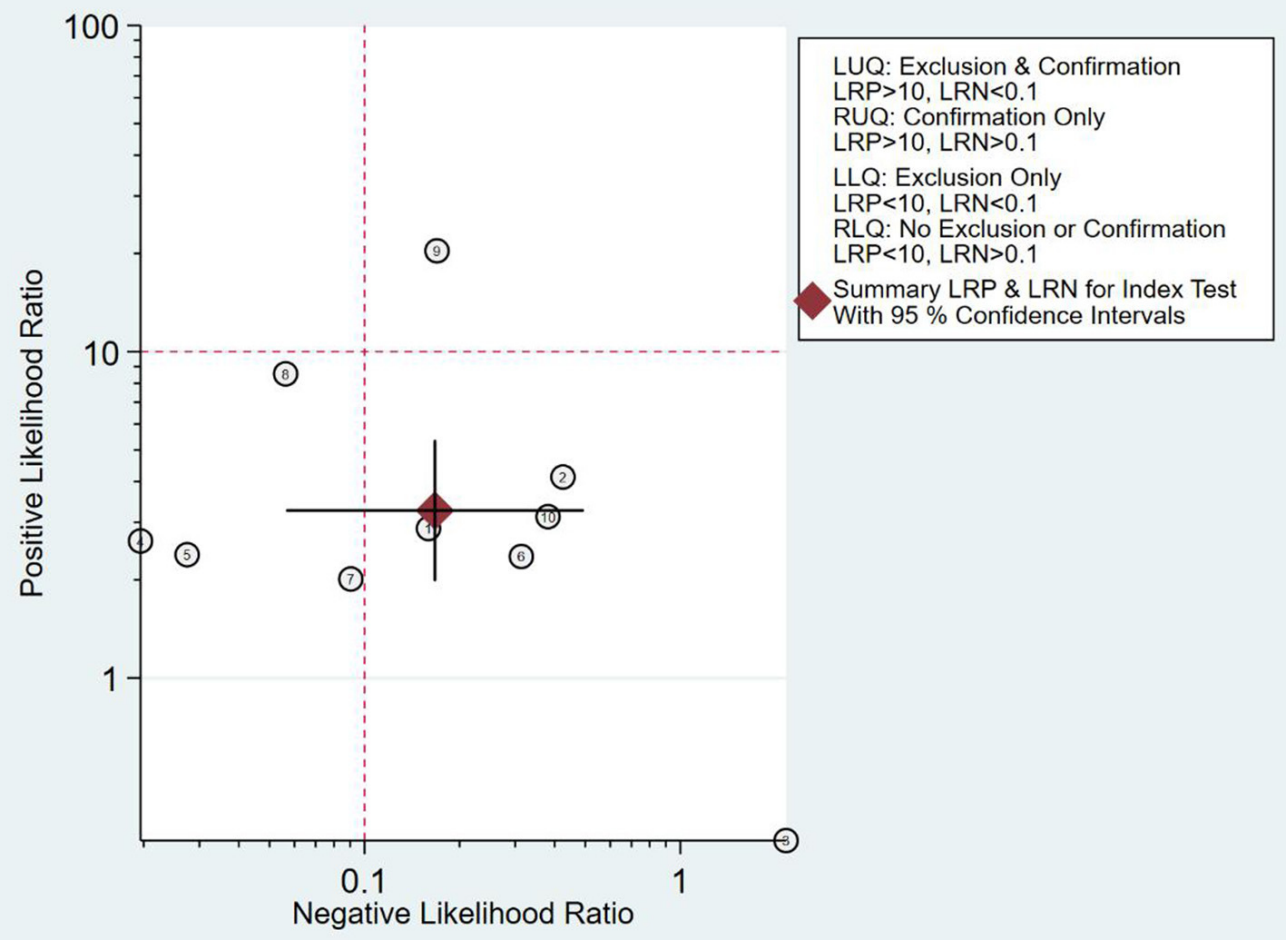
Likelihood ratio dot plot.

**Figure 8:**
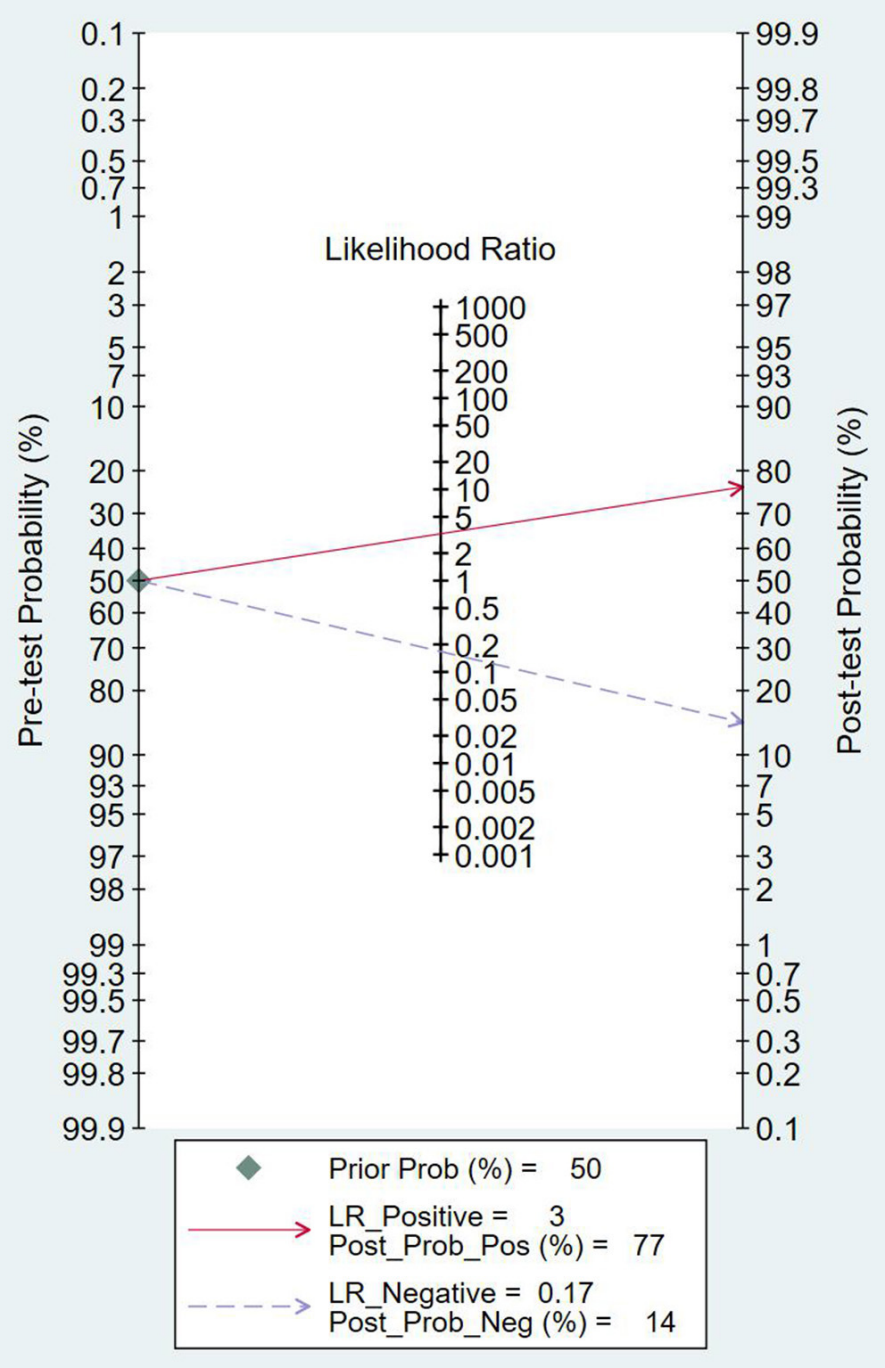
The Fagan map.

As can be seen from Figure 7, the combined negative likelihood ratios for the diagnosis of PCa were >0.1, and the positive likelihood ratio was <10. A Fagan plot was constructed for clinical application analysis, as shown in Figure 8. The posttest probability of 18-FACBC PET/CT was 77% and is higher than the pretest probability, which is 50%, indicating that 18-FACBC PET/CT is effective for the diagnosis of PCa.

#### Publishing Bias

**Figure 9:**
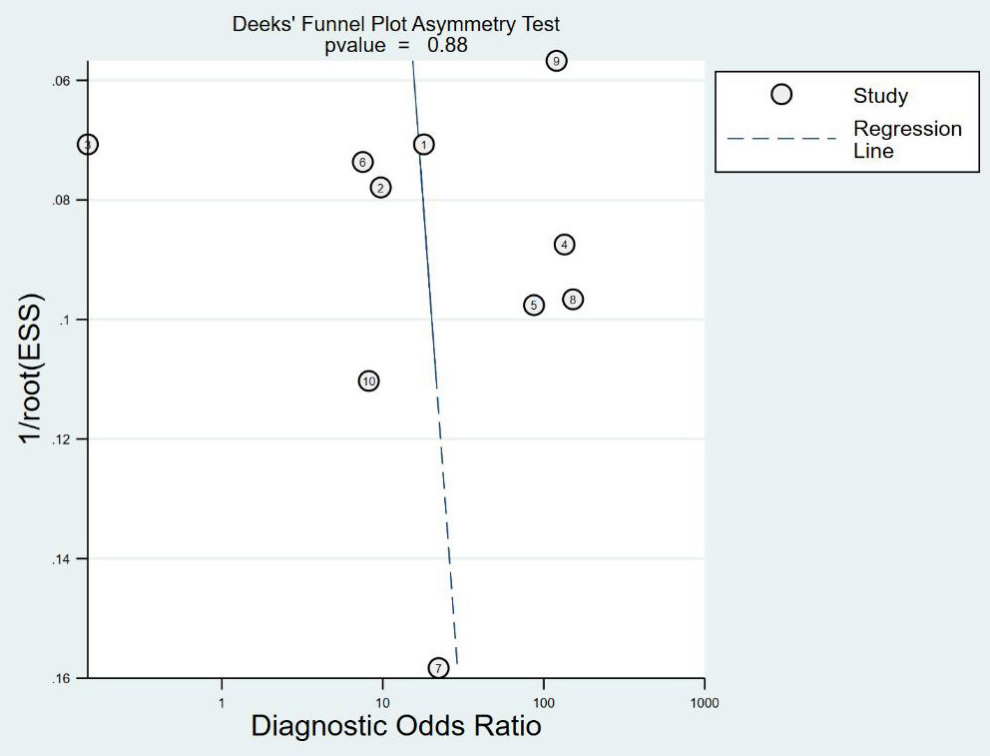
Deek funnel test. ESS, explained sum of squares. Sum of squared deviations that reflect the degree of correlation between the independent and dependent variables.

**Figure 10:**
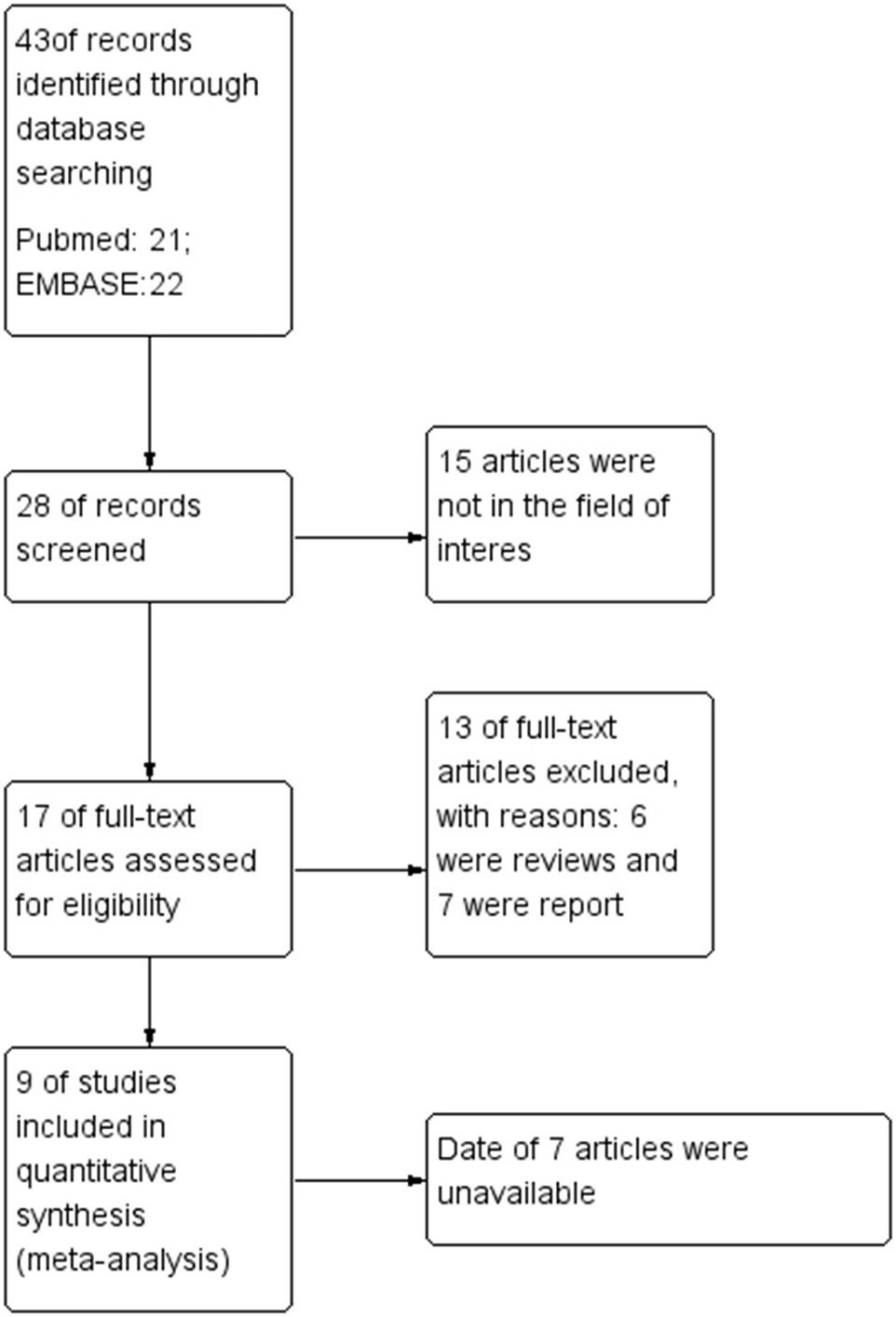
Flowchart.

A Deek funnel plot was drawn, and the symmetry of the funnel was tested by linear regression. The results show that the asymmetry test P-value is 0.088 (>0.05), indicating that the funnel plot is symmetrical and there is no publication bias. It is shown in Figure 9. The flowchart is shown in Figure 10.

## Discussion

18F-FACBC PET/CT fuses anatomical imaging and functional imaging, and it has one-stop inspection features (20). 18F-FACBC is a synthetic amino acid that is not metabolized or incorporated into proteins. It shows increased flow into tumor cells *via* amino acid transporters: alanine-serine-cysteine transporter 2 (ASCT2) and large neutral amino acid transporter (LAT-1). They are upregulated in PCa and show greater impact on progression of castration resistance disease (21–23). 18F-FACBC is excreted slowly through the urinary tract and is less concentrated in the bladder, which is beneficial for imaging. 18F-FACBC PET/CT can evaluate primary prostate tumor lesions and intraprostatic and extraprostatic metastases from analysis of anatomical and functional metabolism (24). The results of this study show that the combined sensitivity and specificity of 18F-FACBC PET/ CT diagnosis of PCa are 88 and 73%, respectively. These suggest that 18F-FACBC PET/CT diagnosis of PCa is accurate. However, the combined negative likelihood ratios for the diagnosis of PCa were >0.1 and the positive likelihood ratio was <10. It seems that 18F-FACBC PET/CT imaging has difficulty in estimating bone metastases (such as in the skull) near the high physiological intake site. The possible cause is that the necrosis and mucus components in the metastases affect the FACBC image, producing a low spatial resolution and partial volume effect.

The heterogeneity in this study is not high. The threshold effect analysis shows that the threshold effect is not the source of heterogeneity. According to the results of the subgroup and meta-regression analyses, the research design and PSA are not sources of heterogeneity. However, due to insufficient data extraction, we only extracted data for four subgroups, and a comprehensive analysis of the source of heterogeneity requires more subgroup data. The 18F-FACBC PET/CT results were interpreted by different methods, including the use of SUVmax value to evaluate 18F-FACBC and the difference in the uptake of 18F-FACBC between the adjacent prostate tissue and the lesion. It also may be the reason for the heterogeneity.

Due to our limited manpower, our search time is from June 2015 to June 2019. However, there are several excellent articles worthy of study. Chung Yao Yu et al. analyzed the effects of five tracers [18F-fluorodeoxyglucose (FDG), 11C-acetate (ACET), 11C- or 18F-choline (CHOL), anti-1-amino-3-18F-fluorocyclobutane-1-carboxylic acid (FACBC), and radiolabeled ligand targeted to prostate-specific membrane antigen (PSMA)]. They find that FACBC has a greater likelihood of detecting local recurrence (25). Jingyun Ren et al. performed a meta-analysis of published data regarding the performance of 18F-FACBC PET/CT in the diagnosis of recurrent prostate carcinoma. 18F-FACBC PET/CT has an 87% pooled sensitivity, 66% pooled specificity on a per patient-based analysis in detecting prostate carcinoma recurrence (26). FDA approves Fluciolovine for screening and detection of recurrent PCa in 2016. And the main study that led to FDA approval of 18F-FACBC is Schuster et al. (27). The liver and pancreas demonstrate the most intense uptake. Although 18F-FACBC exhibited little renal excretion or bladder uptake during the clinically useful early imaging time window, mild to moderate activity might accumulate in the bladder and interfere with evaluation of adjacent prostate bed and seminal vesicles in 5–10% of patients (27). The results of these articles are consistent with ours. 18F-FACBC PET/CT was a non-invasive, metabolic imaging technique in the diagnosis of PCa.

National Comprehensive Cancer Network guidelines consider 18F-FACBC PET-CT for PCa biochemical recurrence localization after radical prostatectomy, whereas European Association of Urology guidelines recommend prostate-specific membrane antigen (PSMA) PET-CT. Calais et al. compared prospectively paired 18F-FACBC and PSMA PET-CT scans for localizing biochemical recurrence of PCa after radical prostatectomy in patients with low PSA concentrations (<2.0 ng/ml). They find that PSMA PET-CT detection rates at the patient level and at the regional level for pelvic lymph node regions and for extrapelvic metastasis were more than twice as high as those for 18F-FACBC PET-CT (28). However, because the PET findings could not be validated by a gold reference standard in two-thirds of patients, neither sensitivity nor specificity could be established. However, in another study, Birgit Pernthaler et al. conducted a prospective head-to-head comparison on 18F-FACBC 68Ga-PSMA-11 in patients with biochemical recurrence of PCa. They demonstrate that 18F-FACBC has a significantly higher detection rate than 68Ga-PSMA-11 (37.9 vs. 27.6%, respectively) for PCa LR occurring in areas close to the urinary bladder (29). The higher LR detection rates are best explained by relatively slow physiological urinary excretion of 18F-FACBC in comparison to 68Ga-PSMA-11. Furthermore, rapid tumor uptake of tracer affording image acquisition occurs shortly after 18F-FACBC administration. This results in negligible activity in the urinary bladder and in a higher tumor-to-background ratio. Nevertheless, further analyses are needed.

There are several limitations of this study. First, comprehensive analysis of heterogeneity sources requires more subgroup data. Second, inconsistent “gold standard” inclusion in the studies may lead to verification bias Third, for the diagnosis of lesions, there are some errors because of the different learning curves and experience of various researchers.

In summary, 18F-FACBC PET/CT has a good diagnostic value for PCa, especially for patients with better diagnostic sensitivity, and it is an effective means for non-invasive diagnosis of liver metastases. However, its diagnostic value in bone metastasis requires more study.

## Conclusion

Based on available literature data, 18F-FACBC PET/CT seems to demonstrate a good diagnostic performance in detecting PCa.

The literature on this topic is still limited, and further investigations on 18F-FACBC PET/CT in patients with PCa are warranted. Based on available data, this imaging method has a good potential, and more studies are needed.

## Data Availability Statement

All datasets generated for this study are included in the article/supplementary material.

## Author's Note

XB attending physician. Medical Expertise: diagnosis and treatment of prostate diseases. Visiting Scholar at the University of Texas Houston Medical Center and MD Anderson Cancer Center. He is a youth member of Nanjing Medical Association's Urology Branch. He won the Jiangsu provincial health planning commission medical new technology introduction award and Scientific Progress Award.

## Author Contributions

XB was responsible for experimental design, experimental analysis and thesis writing. SY, Q-FK, SZ, G-YZ, J-PW, S-QC, and W-DZ were responsible for literature retrieval and data screening. K-HP, M-LD, and MC were responsible for the guidance and review of the thesis.

### Conflict of Interest

The authors declare that the research was conducted in the absence of any commercial or financial relationships that could be construed as a potential conflict of interest.
